# The Effect of Teaching Games of Understanding as a Coaching Instruction Had on Adjust, Cover and Heart Rate among Malaysian and Indian Junior Hockey Players

**DOI:** 10.3390/sports5020044

**Published:** 2017-06-20

**Authors:** Sanmuga Nathan

**Affiliations:** Universiti Pendidikan Sultan Idris, Tanjong Malim, Perak Darul Ridzuan 35900, Malaysia; sanmuga@fsskj.upsi.edu.my

**Keywords:** TGfU, Skill-drill-Technical (SDT), adjust, cover, heart-rate, cardiovascular

## Abstract

The field hockey coaching process across both Malaysia and India favours a traditional, coach-centred approach of mastering technical skills in terms of game play parameters, fitness, intensity, and load training, whereas a tactical- and player-centred pedagogical approach still takes a backseat. On the other hand, the Teaching Games for Understanding (TGfU) model offers tactical-cognitive instruction and is gaining international recognition for its ability to produce intelligent players via a problem-solving approach in game play. Therefore, the purpose of this quasi-experimental study was to investigate the effect of TGfU compared to skill mastery instruction, termed as Skill Drill Technical (SDT), among Malaysian and Indian elite junior hockey players in term of the game play attributes of adjust and cover in 5 vs. 5 small-sided game play and game play intensity via heart rate (HR) at different points of game play. A total of *n* = 60 players with an average age of 15 ± 1.03 was selected via simple random sampling from both countries involved in this study and assigned equally to groups, with 15 per group for TGfU and for SDT across Malaysia and India. Gathered data were analysed using the ANOVA and ANCOVA techniques. Findings indicated that there were no significant differences for adjust in 5 vs. 5 game play between TGfU and SDT across Malaysia and India after the intervention. For cover, there was significant improvement for Malaysian players using the TGfU model compared to SDT. In contrast, there was no significant difference between these two models among the Indian players after the intervention. There was significant difference between these two models in terms of warm-up HR across the two countries, and HR was higher via TGfU. For HR immediately after the 5 vs. 5 game play intervention and HR after three minutes’ recovery, Indian players with TGfU recorded a higher and significant difference compared to SDT. However, findings indicated no significant difference between these two instruction types among Malaysians, although TGfU proved to have higher HR intensity. Therefore, these findings reiterated that TGfU is a useful approach for game play to enhance intensity and cardiac output. In conclusion, for TGfU to be more relevant to the coaching environment, future research should link game play and physiological parameters. TGfU should able to overcome the barriers of tradition and cultural background that may hinder its momentum

## 1. Introduction

Teaching Games for Understanding (TGfU) is a much sought-after game-based pedagogical model in physical education and coaching contexts in Europe, the USA, Canada, Australia, and Asian countries such as Singapore, Japan and Hong Kong [1,2,3]. However, implementing TGfU across Malaysia and India is still problematic. In Malaysia, TGfU is at the early stages of implementation; curriculum planners and coaches much prefer skill-based teaching that gives importance to biomechanics principles and a motor learning teaching approach, whereby tactical elements of decision making and problem solving—the key tenets of TGfU taking backseat. Similarly, the Indian coaching and teaching context, especially in field hockey, is still very much inclined to the skill-based technical model [4]. This similarity in pedagogical approach in coaching between Malaysia and India is probably due to a shared Eastern tradition and cultural background.

In the context of the TGfU model, extensive research has been undertaken on attacking strategy players in terms of ball control, skill execution such dribbling, passing, tactical decision making, supporting players without the ball in small-sided game play such as three vs. three or four vs. four, and so on [1,5]. As it stands today, researchers argue that limited research examines the effects of TGfU on small-sided game play performance in term of defence strategy such as cover and adjust [6,7,8]. 

Another issue is that most scientific work done in TGfU relates to learning outcome and learning process compared to physiological parameters such as intensity, volume, load, and fitness level, which influence mini or small side game play performance and game play configuration. TGfU proponents and coaches should consider game play volume and intensity to improve specific fitness, skilled performance of players. 

On the other hand, skill-based sports coaching research has greatly evolved regarding physiological attributes such as fitness, periodization, and metabolic demands during simulated training matches and competition. Heart rate (HR) and rate of athlete’s perception of effort (RPE) have been thoroughly investigated to determine training intensity [9,10,11]. However, limited study has been undertaken to investigate the effect of TGfU via mini or small-sided game-play intensity evaluated using the crucial HR tool [12,13,14]. Memmert has raised questions on how game coaching development programmes could benefit from the player-centred pedagogical approach of TGfU and how it can be implemented in coach education [15]. Therefore, to answer the questions raised by Memmert, findings from TGfU in the coaching context in terms of mini or small-sided game intensity as physiological attributes may be crucial to determine future milestones of TGfU [2,15,16] and for the TGfU model to improve and develop, within coaching contexts, on the standard and quality of game play. Research should investigate physiological attributes such as volume and intensity along side the game-play components of skill execution and decision making as well as cover and adjust while defending. TGfU research has acknowledged that there is a linear relationship between motor performances of ball control and the acquisition of game knowledge through mini games [17,18]. In addition to ball control and game knowledge relationship, skilled performance correlates with cardio-respiratory endurance and intensity, as in three vs. three small-sided game play [19,20].

Therefore, this paper attempts to answer Memmert and colleagues in their ten research questions pertaining the future of TGfU. TGfU could provide a strong basis for coaches to utilize this approach in training routines to upgrade physiological attributes along with game-play performance [21]. Coaches should also give importance to this pedagogical approach, which can influence physiological attributes of game play intensity and performance [22]. Serious problems will arise when coaches and teachers do not plan their small-sided game play based on intensities. Anecdotal proof indicates that low-intensity small-sided game-play activities will drive away high-skilled players and vice versa [20]. On the other hand, pedagogical approaches such as TGfU, as a non-linear pedagogy practised by teachers, need to be adapted by coaches to help players to build high-order thinking skills as they relate to tactical game problem-solving applications and skill learning. Findings of TGfU instruction have indicated that players are enabled to implement correct game decision making and show improvement in their declarative and procedural game knowledge in the physical education context [22,23,24,25,26]. However, little is currently known, especially regarding cover and adjust in the coaching context.

Research completed via TGfU in physical education and coaching has greatly focused on attacking strategy in terms of tactical decision making and skill execution in game play rather than defence strategy in terms of cover and adjust, which are equally important in game-play performance. To date, only a few research studies have been conducted, mainly in physical education settings rather than coaching. Among notable research are studies done some time ago in soccer by Harvey and his associates, which examine the defensive aspects of off-the-ball cover and adjust among American high-school soccer players [6,27]. Their findings revealed significant changes between the baseline and intervention phases in appropriate adjusts for both teams and inappropriate covers and overall appropriate game performance [6,27]. In another basketball study by Gray and Sproule, their findings used a game-based approach compared to a direct approach; their post-test findings indicated significant improvement by players in off-the-ball game-play performance in examples giving good support [28].

The game of hockey, whether field hockey or ice hockey, is characterized by a high volume of load, anaerobic intensity and cardiovascular fitness as well as the need for good recovery to optimize game-play performance [29,30]. Training volume and load are easily monitored via duration of time spent on activities such as small-sided game play, but other types of intensity are much more difficult to assess. Tools such as such as heart rate (HR), oxygen consumption, weight lifted, blood lactate and rate of athlete’s perception of effort (RPE) during training allow researchers to measure training intensity [12,31]. Small-sided game play, as proposed in TGfU, significantly contributes to higher cardiac intensity, and therefore coaches should monitor this closely via heart rate (HR) or RPE measurements as these tools are handy and can be field-tested. In hockey, to date, limited research has been conducted examining the intensity of small-side game play as proposed in the TGfU model via HR, which is crucial cardiac output. Although various types of electronic heart rate exist, players frequently employ radial and carotid HR measurement to be taken with pre-and post-exercise pulse palpation. This method is the most popular because it is easy and no instrument is needed [32]. A reasonable number of research studies so far have indicated the importance of HR monitoring intensity for activities or training, including small-sided game play, which is an integral part of the TGfU approach [12,33,34]. Findings by Asci indicated 3 vs. 3 recorded higher HR and %HR max compared to 9-a-side game play [12].

The importance of HR measurement is emphasized by Lang and Liu through their findings using small-sided game play of the four vs. four method; after a three-month intervention, female Beijing youth football players improved their average HR of 177 b/min and the maximum oxygen uptake of 94.81 mL/kg [35]. Another study by Sell and Ledesma examined maximum heart rate (MHR) and energy expenditure using ten female field hockey players during competitive play using the Yo-Yo Intermittent Endurance test to determine maximum heart rate. No significant differences in MHR were observed between playing positions, while the other high energy expenditure (kcal) was indicated in heavy-intensity exercise [36]. Stanula and Rocznioke examined twenty Polish junior national ice-hockey players’ playing intensity as low, moderate and high, based on their heart rates (HRs) recorded during a game using the maximum oxygen uptake test to determine intensity zones. Findings indicated the forwards spent more time in the low-intensity zone than the defence men [37]. Therefore, the results of the study indicated that using aerobic and anaerobic metabolism via HR variables to determine intensity zones can be a useful tool for coaches negotiating TGfU in managing the training process. Another study by Cherappurath and Kabeer compared physiological variables between football and handball players at Pondicherry University using *n* = 30 male students, aged 19 to 22 years. Findings indicated no significant difference in resting pulse rate between football players and handball players [38]. Based on these studies, the intensity of training activities that can be monitored by HR is crucial, especially in the coaching context; however, few studies and coaches consider the role that pedagogical approach may have in influencing intensity of training based on their planned activities.

**Figure 1:**
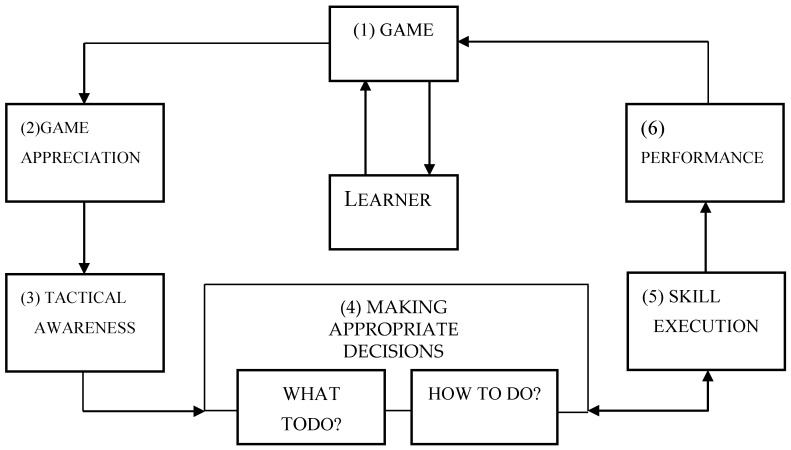
TGfU original model (Bunker & Thorpe, 1982).

TGfU evolved as a practical application of a six-step learning model, as illustrated in Figure 1, practised at Loughborough University in the late 1960s [5,39].Today, TGfU is a frequently pursued non-linear game-learning model compared to linear and structured skills-led models [13,30,40]. The original TGfU model is still relevant with some slight adjustment in terms of providing students with cue perception and skill drills, as suggested in the revised version of TGfU proposed by Kirk and MacPhail [40], as this model caters to a product and process curriculum approach. The traditionally linear and structural model of skill-led pedagogy used structured lessons focusing on skill development and dominated by the coach or teacher [21,33]. The skill-led structural or linear model emphasizes skills development and technical enhancement before players undertake authentic game play [26,41,42]. This approach gives due importance to skill development compared to tactical thinking and tactical game play [43,44]. Even though the skill based-led model seems to be less effective and outdated compared to game-based approaches such as TGfU, the skill-based model still seems to be strong in some countries. Anecdotal evidence indicates that cultural and situated learning perspectives of coaches in Malaysia and India seem to hold as the central tenet that the coach is central, playing important roles such providing a respected role model, cues and feedbacks. 

Based on significant anecdotal evidence and observations, the problem with most coaching approaches in Malaysia and India is that they are carried out using structural lessons and the skill-led technical model, whereby their training units seems to be very structured with warm-up activities, skill demonstration, skill drills and some game play, and limbering down at the end of the training unit. In this research, the researcher labelled this method of coaching the Skill Drills Technical (SDT) model. Based on anecdotal evidence, most Malaysian and Indian hockey players, especially the junior players, are unable to make correct decisions on using appropriate tactics and skills in game situations. Few Malaysian and Indian researchers and coaches have developed, applied and investigated the effectiveness of the nonlinear pedagogy of the TGfU model in their coaching pursuits. As TGfU underpins player-centred instruction, numerous activities can be arranged in simulated game-play situations using tactical-skill problem solving, and coaches or teachers can employ a guided discovery approach to enable players or students to make correct decisions using appropriate tactical-skill elements in the game and to solve the game-play problem.

In relation to the above problem, the purpose of this study was to investigate the effect of TGfU compared to a traditional coaching approach that emphasizes technical development and skill drills, which in this research is labelled as Skill Drill Technical (SDT) and functions as a control group. This study investigates these two approaches in terms of cover, adjust, HR before game play, HR after game play, and recovery after three minutes of 5 vs. 5 game play among Malaysian and Indian elite junior hockey players before and after interventions. The study addresses the following null hypotheses in particular :there was no significant difference between TGfU and SDT for cover and adjust in 5 vs. 5 small-sided game performances among Malaysian and Indian junior elite hockey players before and after training intervention; there was no significant difference between TGfU compared to SDT in terms of HR before game play, after game play, and three minutes after recovery among Malaysian and Indian junior elite hockey players before and after training intervention.

## 2. Methodology

A balanced sample group experimental design with a pre-and post-test control group design was employed in this study to determine the effect of TGfU and SDT pedagogical coaching approaches in 5 vs. 5 hockey game play across Malaysia and India. The effectiveness these two pedagogical approaches was assessed and evaluated in terms of cover, adjust and HR beats/intensity before, immediately after and after three minutes’ recovery after small-side 5 vs. 5 game play among Malaysian and Indian junior elite hockey players before and after training intervention.

### 2.1. Participants

As for participant selection, measures were taken to ensure the participants in both countries were equal in term of baseline performance using various strategies. The participants in both countries should have three years’ experience playing hockey. Participants from both countries should participate in a hockey development programme. Malaysian participants were drawn from on sports school players, while Indian players were drawn from a hockey academy based in New Delhi. Both countries’ participants were under the care of national coaches.

The Malaysian sample consists of *n* = 30 sports school players, aged 15 years ± 1.0, who were selected randomly out of a total of *n* = 45 players; using only 30 in this sample was a limitation of this study. This sample was assigned and distributed equally into groups of TGfU, *n* = 15 and SDT model, *n* = 15. The players had some experience playing hockey using a skill-based approach. Informed consent was obtained from all 30 participants and their coaches, parents or guardians as well as from the Malaysian Ministry of Education. For the Indian players, they also comprised a sample of *n* = 30 players out of a possible 60 from Indian academic junior hockey players (aged 14–16 ± 2.0 years old), who were selected randomly using the simple random sampling technique and assigned equally into groups of TGfU, *n* = 15 and control group SDT, *n* = 15. These players also had some experience playing hockey using a skill-based approach. Informed consent was obtained from all 30 participants and from parents or guardians through their academic coaches based at National Stadium, New Delhi, India. Precautions have been taken to minimize the injury level by stationing qualified physiotherapists to monitor any issues related to injury, in line with ethical principles in dealing human in research.

Two qualified hockey coaches, each with more than 10 years’ coaching experience, were assigned in Malaysia and India to coach these players using the two pedagogical models in coaching contexts. Some measures were taken to control the fidelity in implementation of these pedagogical models in coaching contexts; the following steps were taken. The principal researcher conducted some piloting work, such as consulting experts for validation and testing the intervention models. The principal research conducted a reliability study on the instruments used prior to the actual study. Before the actual study, the principal researcher conducted simultaneous briefings and practical session on the procedures and steps on implementing these two different pedagogical models in their coaching processes to upgrade the players’ hockey game configuration and performance. These coaches from each country were given training units and a log book as well as a checklist on implementing the two pedagogical models in their coaching. Two pre-coaching units each on TGfU and SDT models and procedures, as well as testing dependent variables, were conducted by the principal researcher to help the coaches familiarize themselves with TGfU and SDT model procedures before the actual coaching interventions. Furthermore, discussion and interviews were conducted by the principal researcher to measure and check the reliability of intervention procedures by the selected coaches.

### 2.2. Measurements

#### 2.2.1. Cover and Adjust

This study adopted measurement for adjust and cover from a reliable and tactical approach assessment, “Game Performance Assessment Instrument **(**GPAI)” a game-play observational instrument used with permission from Mitchell, Oslin and Griffin [43,45,46]. Adjust means a movement of the performer or player, either offensively or defensively, determined by the flow of the game. Cover provides appropriate defensive cover, help, or backup for a player making a challenge for the ball or projectile during defensive strategy [46].

The game performances of the dependent variables of cover and adjust were coded 5, 4, 3, 2 for successfully (5-very effective performance; 4-usually effective performance; 3-moderately effective performance, sometimes; 2-very weak performance) and 1 (very weak performance, never) for unsuccessful. A total of two experienced and qualified Malaysian and Indian hockey coaches were trained to code all the dependent variables using the game-play observational instrument by watching two videotapes of 5 vs. 5 game-play situations. For inter-coder reliability, based on the 20 players featured in two game situations of 5 vs. 5, the agreements between the coder and principal researcher were 85% for cover and 84% for adjust.

#### 2.2.2. Radial Heart Measurement

Even though there are many types of intensity measuring tools available, for this research, radial HR was employed to measure intensity at different points of small-sided hockey game play. The most popular post-exercise pulse palpation utilized radial (wrist) heart rate measurement for 6 s (number HB in 6 s × 10 to get HR per minute (HRM)) because it is relatively easy and requires no equipment [33]. In this research, to measure HR or pulse rate, the Wrist Radial palpitation method was used to take the pulse at three different point intervals in order to measure HR before and after small-sided game play of 5 vs. 5, and another HR was collected after 3 min of game play to determine recovery HR. In detail, the following interval HRs were measured: (a) before small-sided game play of 5 vs. 5, (b) immediately after 5 vs. 5 small game play, and (c) final interval HR (recovery HR) after 3 min of 5 vs. 5 small-sided game play to detect the rate of players’ recovery. The pulse was taken for 10 s × 6 (for a 1-min pulse rate reading). Meanwhile, participants were given three trials of HR measurement before the actual study to make them comfortable with the radial palpation technique.

### 2.3. Coaching Intervention

As for intensity, volume and load, the selected players in the TGfU and SDT intervention groups underwent three (3) training or coaching sessions per week for two (2) h per session for five weeks. Both groups in Malaysia and India followed similar activities in terms of volume (2 h per session), intensity (low intensity at 120–140 HR, medium intensity at 130–150 HR and high intensity at 150–170 HR). For rest, active three (3) rest were given from one activity to another activity.

The TGfU group predominantly trained their tactics, skills, fitness and physical conditioning components via small-sided game situations as the main coaching activities to improve their related game-play skills in game-based tactical thinking and solving game-related strategies of attacking and defending in game-play situations. Briefly, the TGfU coaching process encourages the players to discuss and think by answering questions and solving game-play problems through practical application. Questions are based on ‘what to do’ and ‘how to do’ in terms of manipulating tactics and proper skill execution in game play. The TGfU approach helps the players decide on and apply appropriate tactics and skills in small-sided game situations and activities [13,47]. The daily coaching unit revolved around the following phases: phase 1,general game play discussion, specific warm-up session (game play for 15 min at 120–140 low-intensity HR); phase 2, short guided discussion, questions and discussion on tactics on attacking and defending strategy, followed by application of discussed tactics in small-sided game play I, (game play for 20 min at 130–150 medium-intensity HR); phase 3, another short briefing, discussion, and application pf skills, followed by game play II (game play for 20 min at 150–170 high-intensity HR); phase 4, activities limited to limbering down and reflection-feedback activities (5 min).

On the other hand, SDT coaching approaches players by means of linear instruction conducted by coaches, which in terms of volume and intensity as well rest is similar to the TGfU approach, but focuses on skill teaching and learning, mastery skill employing, skill drills activities and feedback as the main content of coaching. Players are also given some form of game play, either small-sided game play or full game play, allocated at the end of each coaching unit. The implementation of these two intervention periods is based on sports training principles and motor learning principles [4,48]. 

### 2.4. Research Conceptual Framework

**Figure 2:**
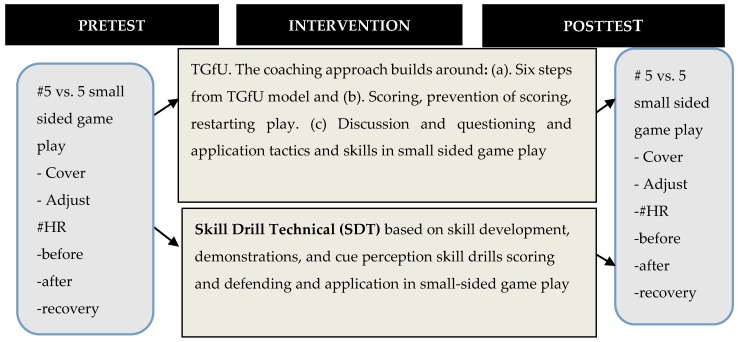
Research Conceptual Framework.

The research conceptual framework, illustrated in Figure 2, focused on TGfU and SDT as hockey coaching approaches. The TGfU hockey coaching content was delivered via the six teaching steps proposed in the original TGfU model [13]. The organization of the coaching activities predominantly utilized small-sided game play approach, in which the players were involved in various small-sided game plays and discussion and questioning occurred with regards to application of tactics and skills. Tactics applied in the TGfU model underpinned scoring strategy, prevention scoring, and restarting game-play strategy based from the framework of Mitchell [47]. On the other hand, in the SDT coaching approach, the contents and coaching implementations revolve around skills development, demonstrations, and cue perception skill drills activities and feedback, as well as small-sided or full-sided game play at the end of the coaching unit. The effectiveness of these coaching approaches was evaluated in terms of cover, adjust and HR measurement before and after small-sided game play situations and after three minutes’ recovery before and after coaching intervention among hockey players across Malaysia and India.

### 2.5. Data Collection and Analysis

Parameters of adjust and cover in 5 vs. 5 small-sided game play were calculated with total marks using rubrics marks from 5-1on a level of successful responses. As HR responds to warm-up rate, after mini-game activities and recovery HR, pre- and post-tests were calculated based on total number of pulses per minute. Pre-test data was screened for normality using the Kolmogorov-Smirnov and Shapiro-Wilk tests for all dependent variables; the findings of both tests indicated no significant difference with *p* > 0.05, hence, normality of data prevailed. Data from all the dependent variables from pre-test and post-test were analysed through ANOVA using SPSS software version 21. Furthermore, the inferential statistic of ANCOVA was employed to confirm the results when significant difference were yielded at the pre-test level. 

## 3. Results

### 3.1. Adjust

**Figure 3:**
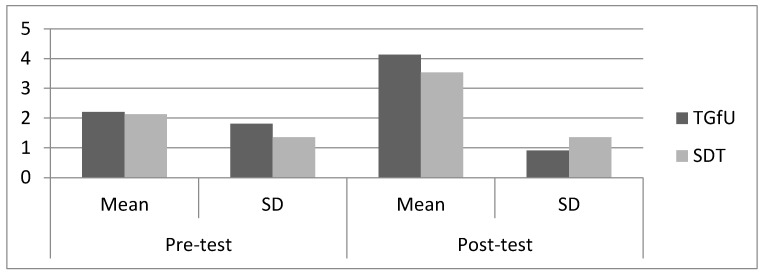
Pre-test and post-test result for adjust among Malaysian players.

Pre-test results indicated there was no significant difference between the TGfU (2.20 ± 1.81) and SDT approaches (2.13 ± 1.35) for adjust, *F*(1,28) = 0.013, *p* > 0.05 among Malaysian players. For post-12 test results, there was also no significant difference between TGfU (4.13 ± 0.91) and SDT (3.53 ± 1.35), *F*(1,28) = 2.02, *p* < 0.05 for adjust. Figure 3 illustrates the mean and SD pre-test and post-test results for adjust among Malaysian players.

**Figure 4:**
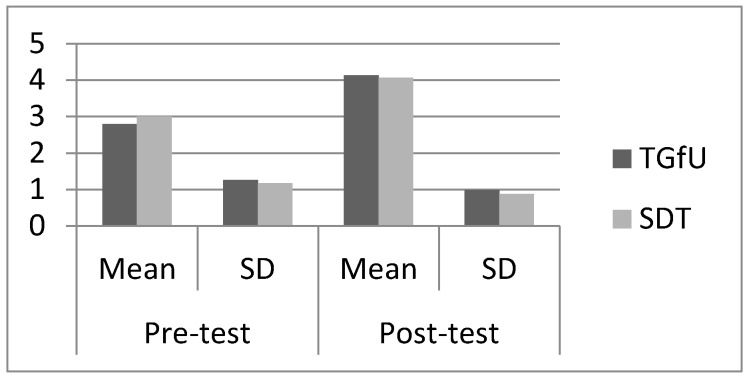
Pre-test and post-test result for adjust among Indian players.

As for the Indian hockey players, pre-test results indicated there was also no significant difference between the TGfU (2.80 ± 1.26) and SDT approaches (3.60 ± 1.18) in adjust, *F*(1,28) = 3.20, *p* > 0.05 before intervention. Post-test results also indicated there was no significant difference between TGfU (4.13 ± 0.99) and SDT (407± 0.884), *F*(1,28) = 0.038, *p* > 0.05. Figure 4 illustrates the mean and SD pre-test and post-test results for adjust among Indian players.

### 3.2. Cover (Mark)

**Figure 5:**
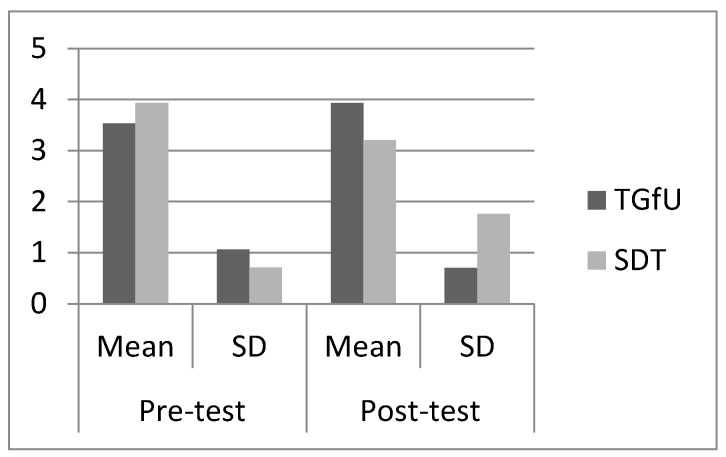
Pre-test and post-test result for cover among Malaysian players.

For cover, pre-test findings indicated there was no significant difference between TGfU (3.53 ± 1.06) and SDT (3.93 ± 0.71), *F*(1,28) = 1.48, *p* > 0.05 among Malaysian players. Post-test results for cover recorded a significant difference between TGfU (3.93 ± 0.70) and SDT (3.20 ± 1.76), *F*(1,28) = 7.36, *p* < 0.05 for Malaysian players. Figure 5 shows the mean and SD pre-test and post-test results for cover among Malaysian players.

**Figure 6:**
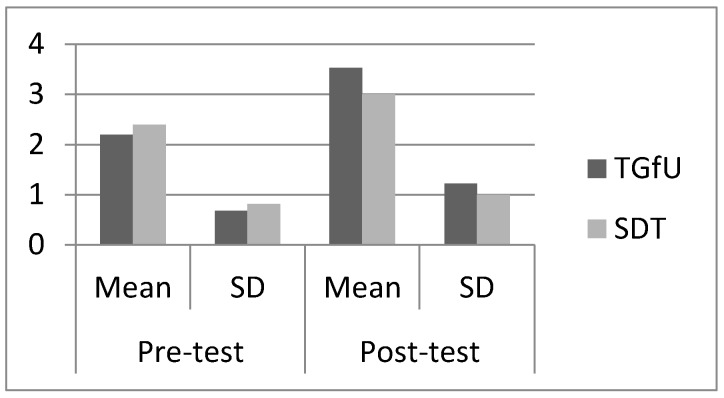
Pre-test and post-test result for cover among Indian players.

As for Indian players, pre-test findings on cover indicated there was no significant difference between TGfU (2.20 ± 0.68) and SDT (2.40 ± 0.81), *F*(1,28) = 0.525, *p* > 0.05 among the players. For post-test results, there was also no significant difference between TGfU (3.53±1.22) and SDT (3.00 ± 1.00), *F*(1,28) = 1.77, *p* > 0.05. Figure 6 illustrates the mean and SD pre-test and post-test results for cover among Indian players.

### 3.3. Heart-Rate (HR)

As indicated by Table 1 below, the following statistical results were recorded for Malaysian players in term of HR. For pre-test, warm-up HR before intervention indicated no significant difference between players in TGfU (82.07 ± 4.46) and SDT (84.1 ± 4.04), *F*(1,28) = 1.65, *p* > 0.05. In contrast, the post-test result for warm-up HR before small-sided game play indicated a significant difference between TGfU (78.86 ± 2.87) and SDT (77.60 ± 3.45), *F*(1,28) = 5.27, *p* < 0.05. Pre-test HR immediately after small-sided 5 vs. 5 game play indicated no significant difference between TGfU (135.20 ± 6.70) and SDT (130.60 ± 4.11), *F*(1,28) = 0.220, *p* > 0.05. Post-test HR immediately after small-sided 5 vs. 5 game play also revealed no significant difference between TGfU (126.26 ± 5.68) and SDT (128.20 ± 5.44) among Malaysian players, *F*(1,28) = 1.87, *p* > 0.05. For recovery HR after three minutes, pre-test results indicated no significant difference between TGfU (82.46 ± 4.45) and SDT (83.20 ± 4.10) among Malaysian players *F*(1,28) = 0.906, *p* > 0.05. Post-test results for HR after three minutes also indicated no significant difference between the TGfU (79.33 ± 4.20) and SDT (80.13 ± 4.58) pedagogical models, *F*(1,28) = 0.906, *p* > 0.05.

As indicated by Table 2, below, the following results were recorded for Indian hockey players in terms of HR findings. The pre-test warm-up HR before the intervention phase indicated no significant difference between players in TGfU (83.80 ± 6.96) and SDT (84.26 ± 3.61), *F*(1,28) = 0.053, *p* > 0.05. However, the post-test result for warm-up HR indicated significant difference detected between TGfU (82.66 ± 6.21) and SDT (77.86 ± 3.22), *F*(1,28) = 7.04, *p* > 0.05. For pre-test HR, immediately after 5 vs. 5 game play indicated significant difference between TGfU (134.80 ± 5.78) and SDT (128.40 ± 4.48), *F*(1,28) = 11.46, *p* < 0.05. Post-test HR immediately after 5 vs. 5 small-sided game play revealed significant difference between TGfU (136.73 ± 5.68) and SDT (130.60 ± 4.11) among Indian players, *F*(1,28) = 8.80, *p* < 0.05. Therefore, to confirm this result, ANCOVA analysis was carried out as stipulated in Table 3 and Table 4, and the findings confirmed that there was a significant difference between these two models after intervention in terms of HR immediately after 5 vs. 5 game play. For recovery HR after three minutes of 5 vs. 5 small-sided game play, pre-test results indicated no significant difference between TGfU (87.00 ± 5.35) and SDT (85.06 ± 4.09) among Indian players *F*(1,28) = 1.22, *p* > 0.05. However, post-test results for HR after three minutes of 5 vs. 5 small-sided game play results indicated significant difference between the TGfU (100.53 ± 4.38) and SDT (95.73 ± 6.95) pedagogical models, *F*(1, 28) = 5.11, *p* > 0.05.

This result was confirmed using analysis covariate (ANCOVA), which also indicated significant difference between these two models immediately after game play, *F*(1,27) = 5.05, *p* < 0.05. The results of ANCOVA are presented in Table 3 and the estimated marginal means for HR immediately after play at post-test are presented in Table 4.

## 4. Discussion and Implications

The findings for the component of adjust in 5 vs. 5 game play indicated no significant difference between the TGfU and SDT approaches among Malaysian and Indian players. However, based on mean score, TGfU could be a better model, although it will require more extensive research. The present findings on adjust were in line with previous findings in soccer, hockey and badminton [26,27,39,49,50]. On the other hand, for cover, our findings indicated significant improvement using TGfU among Malaysian players. These findings support the significant findings of Harvey, Light & Fawns and Turner & Martinek [26,27,49,50]. The findings of adjust and cover defensive off-the ball performance are similar to the findings of Harvey and associates, whose university team improved on some of the individual aspects of defensive off-the-ball performance associated with the TGfU intervention [6,27]. Even though this study reports on adjust and cover attributes of game play, findings via TGfU are still inconclusive in the coaching environment across Malaysia and India, as these two countries are very much influenced by tradition and a background of coach-centred and skill-based philosophy. Perhaps a partnership with ecological learning theory from the motor learning perspective, which focuses on skill development, may support the adoption of the TGfU model as the global coaching pedagogy.

As for pre-test warm-up intensity, HR indicated no significant difference between the TGfU and SDT models among players in both Malaysia and India, as TGfU and SDT both recorded a higher warm-up HR, probably due to low levels of fitness. There was a slightly lower significance between the two models in terms of mean HR in both countries at post-test mean warm-up HR. However, the TGfU model recorded significantly higher warm-up HR compared to SDT in both countries after intervention. This was probably due to higher intensity warm-up activities set by the TGfU model. On the other hand, findings for HR immediately after small-sided5 vs. 5 game play indicated a significant difference between these models only for Indian players using TGfU (136.73 ± 5.68) compared to Malaysia players with a higher HR. The present findings are similar to findings by Asci, which indicated that 3 vs. 3 recorded higher HR and %HR max compared to 9-a-side game play [12]. This is probably because Indian hockey utilize dribbling and stick-work styles compared to Malaysian players, who play using a passing approach and limited higher intensity of movement even when using the TGfU approach. The findings for Indian players in this research are in line with the findings of Ghosh, Goswami, Mazumdar, and Mathur, whose study indicated that junior hockey players’ (*n* = 25; 18 ± 0.6 years) mean heart rate during a full hockey match was 143.4 [14,51]. Therefore, the TGfU model, through small-sided game play, maximized physiological adaptations for Indian hockey, similar to distance runners’ exercise programmes [52,53]. Like for the distance runner, game-play activity requires slightly high intensity adaptation for better performance. If the intensity is too low, players will not attain superior game-play performance. In comparison, if game-play exercise intensity is too high, players will suffer from fatigue and hampered physiological adaptations [54].

These findings are less similar to findings by Capranica and associates, who found HR values exceeding 170 beats min^−1^ represent high-intensity work activities [55]. If this is the case, then our HR results suggest that the young players participating in both small-sided games should work at higher levels of intensity. The HR findings in this study after intervention recorded lower HR compared to findings by Clemente & Rocha [56], who recorded higher HR at two phases of 2 vs. 2 small-sided game play (171 beats/min and 177 beats/min), while 4 vs. 4 recorded 159 beats/min and 167 beats/min. The present findings revealed that Indian players recorded high heart rates compared to the Malaysian players in small-sided game play. Furthermore, support from videotaped game-play clip analysis indicated that Indian players played at higher intensity of small-sided games compared to Malaysian players.

Post-test results for Malaysian players after three minutes’ recovery recorded lower HR using TGfU (79.33 ± 4.20) and SDT (80.13 ± 4.58) compared to HR immediately after game play. On the other hand, the Indian players’ recovery HR also indicated reduced HR compared to HR immediately after game play. However, Indian player recovery HR recorded higher HR for the TGfU group (100.53 ± 4.38) and the SDT (95.73 ± 6.95) compared to Malaysian players’ recovery HR. These findings indicate that younger players can tolerate and reduce HR reading during the recovery period after a small-sided game of 5 vs. 5 among both Malaysian and Indian hockey players. The present findings illustrate that the TGfU style of game play increased HR findings among both Malaysian and Indian players, which is important for blood circulation and improving players’ physiological systems. However, this research finding indicates that Indian players are able to increase their HR and are slower in recovery compared to Malaysian players due to tradition and background style of playing, especially with the Indian players employing dribble and stick-work techniques and tactics with many movement skills and ball possession tactics in small-sided game play. In contrast, Malaysian players utilize hit-and-run playing tactics that do not increase game play intensity as much. However, based on Indian players’ style of playing, there is significant influence and support through HR measures for TGfU that emphases small-sided game play being able to sub-maximize game-play intensity. The present findings are in line with findings by Mclean and associates, who indicated that number of players in small-sided game play and game format do influence physiological and physical intensity demands differently, hence HR is affected by this influence [21]. In line with findings by Castellano and associates [57], there was higher HR for 3 vs. 3 than 5 vs. 5 games, there being no difference with respect to 7 vs. 7 game play [8]. 

## 5. Conclusions

In conclusion, there was significant improvement for Malaysian players using the TGfU model compared to SDT for cover. In contrast, there was no significant difference between these two models among the Malaysian and Indian players for adjust after the intervention. Findings for post-test warm-up intensity HR performances indicated significant difference between these two models across players in two countries. For HR immediately after mini-game intervention and HR after three minutes’ recovery, findings among Indian players indicated a significant difference between the two pedagogical models compared to Malaysian players. Based on the present findings, TGfU, through small-sided game play, is able to enhance intensity and cardiac output of the players during game play.

Future research should address the importance of monitoring intensity via HR or RPE while implementing pedagogical models such as TGfU or skill-based models. The instructional model does influence physiological and physical attributes such as intensity and volume of any game play, especially in negotiating small-sided game play. In conclusion, for TGfU to be more relevant, future research must address linking game play and physiological parameters. Caution when negotiating TGfU, especially tradition and cultural background, may hinder TGfU from future implementation as a holistic coaching instruction.

## Figures and Tables

**Table 1:** HR (Mean/SD) for phases of activities for Malaysian junior players.

Model/Phase	TGfU	SDT	Significant
Pre-test warm-up HR before game play	82.07 ± 4.46	84.1 ± 4.04	*F*(1,28) = 1.65, *p* > 0.05
Post-test warm-up HR before game play	78.86 ± 2.87	77.60 ± 3.45	*F*(1,28) = 5.27, *p* < 0.05
Pre-test HR immediately after game play	135.20 ± 6.70	130.60 ± 4.11	*F*(1,28) = 0.220, *p* > 0.05
Post-test HR immediately after game play	126.26 ± 5.68	128.20 ± 5.44	*F*(1,28) = 1.87, *p* > 0.05
Pre-test HR after 3 min recovery game play	82.46 ± 4.45	83.20 ± 4.10	*F*(1,28) = 0.906, *p* > 0.05
Post-test HR after 3 min recovery game play	79.33 ± 4.20	80.13 ± 4.58	*F*(1,28) = 0.248, *p* > 0.05

**Table 2:** HR (M/SD) phase of activities for Indian junior players.

Model/Phase	TGfU	SDT	Significant
Pre-test warm-up HR before game play	83.80 ± 6.96	84.26 ± 3.61	*F*(1,28) = 0.053, *p* > 0.05
Post-test warm-up HR before game play	82.66 ± 6.21	77.86 ± 3.22	*F*(1,28) = 7.04, *p* < 0.05
Pre-test immediately game play	134.80 ± 5.78	128.40 ± 4.48	*F*(1,28) = 11.46, *p* < 0.05
Post-test immediately game play	136.73 ± 5.68	130.60 ± 4.11	*F*(1,28) = 8.80, *p* < 0.05
Pre-test after 3 min recovery game play	87.00 ± 5.35	85.06 ± 4.09	*F*(1,28) = 1.22, *p* > 0.05
Post-test after 3 min recovery game play	100.53 ± 4.38	95.73 ± 6.95	*F*(1,28) = 5.11, *p* < 0.05

**Table 3:** Analyses of covariance summary for HR immediately after 5 vs. 5 game play.

Source	Sum of Square	*df*	Mean Square	*F*	Sig
Group	128.183	1	128.183	7.98	0.10

**Table 4:** Estimated marginal means for immediately after 5 vs. 5 game play.

Programme	*Mean*	*SE*	95% Confidence Interval	
			Lower Bound	Upper Bound
TGfU	133.97 ^a^	1.40	131.00	136.84
SDT	129.22 ^a^	1.40	126.35	132.100

^a^ Covariates appearing in the model are evaluated at the following values: game play = 133.30.
